# Treatment of cardiac synovial sarcoma: experience of two cases

**DOI:** 10.1186/s13019-018-0771-0

**Published:** 2018-07-03

**Authors:** Antonella Coli, Giovanni Alfonso Chiariello, Mariangela Novello, Christian Colizzi, Massimo Massetti

**Affiliations:** 1 Institute of Anatomic Pathology, Catholic University of Sacred Heart, Largo F. Vito 1, 00168 Rome, Italy; 2 Department of Cardiovascular Sciences, Catholic University of Sacred Heart, Rome, Italy

**Keywords:** Synovial sarcoma, Cardiac tumors, Therapy, Total artificial heart implantation

## Abstract

**Background:**

Primary heart sarcomas are exceedingly rare tumors. Among primary cardiac sarcomas, synovial sarcoma is one of the rarest, involving cardiac cavities or pericardium.

**Case presentation:**

Two cases of synovial sarcoma are presented with the clinical course and therapy. Both cases were treated with surgery and chemo/radiotherapy. Interestingly, one of the patient, a 52-year-old male with an intracardiac synovial sarcoma, undergone a SynCardia total artificial heart implantation, but died for multiple pulmonary metastases waiting for transplantation.

**Conclusion:**

Complete surgical resection of cardiac synovial sarcoma is the gold standard of therapy, though rarely possible. Although guidelines for the treatment are not well established, due to limited number of cases reported, chemotherapy and radiotherapy are frequently administered and seem to prolong mean patient’s survival. Cardiac transplantation could be considered in selected cases.

## Background

Synovial sarcoma is a rare soft tissue malignant tumor, usually affecting children and young adults [1]. Primary cardiac synovial sarcoma (CSS) is an exceedingly rare heart sarcoma, involving either the pericardium or chambers, with a striking male predominance, prevalently in the first decades [2, 3]. Histologically, synovial sarcoma can display a monophasic, spindle cell pattern, or a biphasic pattern, with glandular structures and spindle cells present in various proportions. In addition to histological characteristics, molecular demonstration of SYT-SS1 or SYT-SSX2 fusion transcript is essential in confirming the diagnosis [4].

Patients present with nonspecific symptoms, including dyspnea, thoracic pain and pericardial effusion. Complete resection, when feasible, is the treatment of choice, with additional chemotherapy and/or radiation therapy, when necessary [2, 5–7]. However, the extreme rarity of the disease, with only about sixty cases described, hampers the establishment of guidelines for the treatment. We hereby report clinico-pathological features and therapy in further two cases.

## Case presentation

### Case 1

**Fig. 1:**
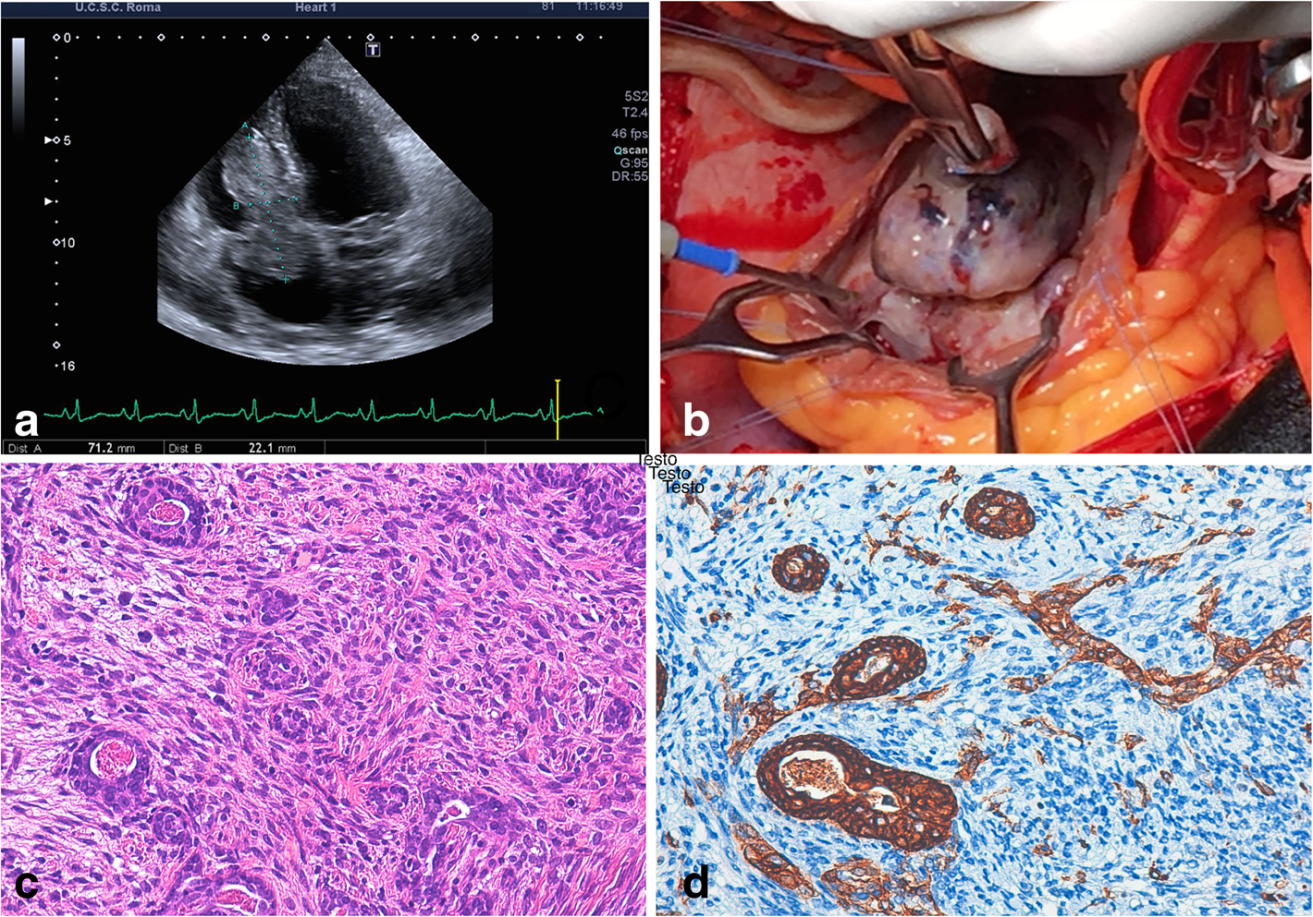
Primary intracardiac synovial sarcoma (case 1). **a** Color Doppler echocardiography identifies a mass adherent to the right side of interventricular septum, projecting into the right ventricle and reaching the right atrium throughout the tricuspid valve. **b** Intraoperative view showing the intraatrial tumor component. **c** Histology revealing a biphasic synovial sarcoma, characterized by glandular structures and spindle cells (20×). **d** Immunohistochemistry showing reactivity for cytokeratin mainly restricted to glands (AE1/AE3, 20×)

A 52-year-old male presented with severe headache and elevated blood pressure (180/120 mmHg). Color Doppler echocardiography revealed a large, solid right intraventricular mass of 7.1 × 2.2 × 4.7 cm, originating from the middle-inferior ventricular septum and extending into the right atrium, with systo-diastolic fluttering (Fig. 1a). A cardiac magnetic resonance imaging (MRI) study confirmed the presence of the solid, intracavitary mass.

A cardiac biopsy via catheterization was not conclusive regarding the precise nature of the lesion. After informed consent, the patient underwent surgery. Intraoperatively, a whitish mass, 3 × 4 cm, non-adherent to the wall, was found in the right atrium after median sternotomy and atriotomy (Fig. 1b). Through the tricuspid valve, the mass extended without discontinuity into the right ventricle, adhering to the septal surface of the leaflet and infiltrating the adjacent interventricular septum, in its medial and superior portion. The exophytic intraatrial and intraventricular components were removed. Since intraoperative frozen-section on tissue from the infiltrated septum confirmed the clinical suspect of malignancy, no further surgery was attempted. Definitive histological examination showed a malignant neoplasm characterized by epithelial cells, positive for cytokeratin, forming glandular structures, admixed with a spindle cell component (Fig. 1c-d). A final diagnosis of biphasic synovial sarcoma was also confirmed by molecular demonstration of an SYT-SSX1 transcript.

Computed tomography scan (CT) and cardiac MRI, performed one month after surgery, prompted administration of chemotherapy (ifosfamide/mesna, 4 cycles/3 months) due to increase of the residual ventricular tumor. However, since at the end of this treatment a (18) F-fluorodeoxyglucose positron emission tomography/CT revealed an increased ventricular mass, a SynCardia total artificial heart was implanted in the patient. Unfortunately, waiting for transplantation, the patient developed multiple pulmonary metastases and rapidly died, one and half year after initial surgery.

### Case 2

A 39-year-old man presented with signs of cardiac tamponade. The echocardiography revealed abundant pericardial effusion with a large intrapericardial mass. The patient underwent surgery after informed consent. At surgery, a pericardial tumor (9.8 × 6.1 × 3.2 cm) adherent to the anterior wall of the aorta, superior vena cava and right atrium, was extensively removed. No deep infiltration of the myocardium was apparent and aortic reconstruction was unnecessary. Since intraoperative histological examination did not reach a conclusive diagnosis, further surgery was not performed. Definitive histology revealed a monophasic (spindle cell) synovial sarcoma carrying an SYT-SSX2 transcript.

Postoperatively, echocardiography, cardiac MRI and CT did not show residual tumor. Owing to a diagnosis of synovial sarcoma, the patient underwent chemotherapy (epirubicin/ifosfamide, 6 cycles/4 months), remaining in healthy conditions for the following 8 months, before the onset of tachycardia and dyspnea. Echocardiography, cardiac MRI and CT revealed a recurrent pericardial tumor extensively involving both atria and left pulmonary veins. Because further surgery was considered unfeasible, an additional course of chemotherapy was started (cisplatinum/docexatel, 6 cycles/4 months), followed by intensity-modulated radiotherapy (DT 54 Gy/25 sessions/2 months). After a temporary improvement of the clinical conditions, CT documented an extensive regrowth of the tumor, with involvement of the heart, great vessels, left bronchus and esophagus, with mediastinal lymphadenopathy. The patient died 32 months after surgery.

## Discussion and conclusion

Cardiac synovial sarcoma is a very uncommon cardiac sarcoma. There is a lack of accurate guidelines for the treatment of CSS owing to the heterogeneity and scarcity of clinical information, derived from a limited number of cases reported on the topic, mainly as single case report. In a recent extensive analysis of 60 CSS patients derived from 54 articles present in the literature, Wang et al. found that overall survival of CSS patients was affected by patient’s age (less than 30 years) and chemotherapy [2]. However, in their analysis, the overall patient’s outcome was poor, due to frequent relapses and metastases.

Indeed, a complete resection of the cardiac tumor is frequently impossible to perform, because of the infiltrativeness and extension of the neoplasm. On the other hand, it is difficult to establish precise chemo-radiotherapy guidelines, due to different adjuvant therapy options in the few cases hitherto described. Hence, the choice of optimal treatment in the single patient is challenging. Therefore, it seems worth investigating both optimal surgical procedures and additional chemo-radiotherapy protocols in order to improve patient’s survival. As a matter of fact, total artificial heart implantation, as performed in one of our patients, could offer a temporary solution looking forward to heart transplantation [8]. Although patients with synovial sarcoma are at risk of pulmonary metastasis, metastasectomy is a good option in metastatic synovial sarcoma, as assessed by Lee et al. in a recent study on fifty patients [9]. Contrasting results have been reported regarding heart transplantation for the treatment of primary cardiac sarcoma. Despite the fact some authors [10, 11] did not find improvement of survival rates in comparison with conventional treatment, other authors [12] have discovered a more prolonged survival after transplantation, in a number of patients.

Bergh et al. [13] found that extra-cardiac synovial sarcoma when occurs in patients less than 25 years, is small in size and presents no undifferentiated areas, has a low-risk of metastases. The above results appear to be confirmed by Wang’s et al. [2] analysis on cardiac synovial sarcoma, since they found a more favorable outcome in patients aged less than thirty years. Therefore, a possible therapeutic option for small intracardiac synovial sarcomas occurring in young patients can be adjuvant chemo-radiotherapy followed by total artificial heart implantation and subsequent transplantation. In this respect, a role for heart transplantation for lower grade and less aggressive cardiac sarcomas has recently been advocated by Li et al. [14].

Thus, as little information on CSS treatment is available in literature, analysis of a major number of cases is necessary in order to establish appropriate therapy protocols. Although limited, our contribution provides further data on the management of this rare malignant tumor.

## Abbreviations

CSS: Cardiac synovial sarcoma
CT: computed tomography scan
MRI: magnetic resonance imaging
